# The Effects of Two Different Rest Intervals on the Repeated Skating Ability of Ice Hockey Forwards and Defensemen

**DOI:** 10.2478/hukin-2022-0102

**Published:** 2022-11-08

**Authors:** Jakub Baron, Subir Gupta, Anna Bieniec, Grzegorz Klich, Tomasz Gabrys, Andrzej Szymon Swinarew, Karel Svatora, Arkadiusz Stanula

**Affiliations:** 1 Institute of Sport Sciences, Jerzy Kukuczka Academy of Physical Education, Katowice, Poland; 2 Faculty of Medical Sciences, University of West Indies, Cave Hill Campus, Barbados; 3 Zagłębie Sosnowiec Hockey Club, Sosnowiec, Poland; 4 Department of Physical Education and Sport Science, Faculty of Pedagogy, University of West Bohemia, Pilsen, Czech Republic; 5 Faculty of Science and Technology, University of Silesia in Katowice, Chorzów, Poland; 6 Department of Kinanthropology and Humanities, Faculty of Physical Education and Sport, Charles University, Prague, Czech Republic

**Keywords:** blood lactate concentration, rate of perceived exertion, skating speed, heart rate

## Abstract

The purpose of this study was to evaluate the effects of two different rest intervals (2 min and 3 min), between two consecutive sets of repeated sprint skating ability (RSSA) tests, on the repeated sprint ability of ice hockey Forwards and Defensemen. Two protocols of RSSA tests, RSSA-2 and RSSA-3, were completed by 16 ice hockey Forwards and 8 Defensemen. Defensemen were heavier (p < 0.05) than Forwards, although their % body fat did not differ significantly. In RSSA-2, athletes performed six sets of 3×80 m sprint skating with 2 min passive recovery between two consecutive sets. In RSSA-3, the rest interval between the sets was 3 min. Average speed, average heart rate (HR_aver_), blood lactate concentration ([BLa]), and the rate of perceived exertion (RPE) were measured in both RSSA-2 and RSSA-3 tests. Both Forwards and Defensemen skated faster in RSSA-3 than in the corresponding set of RSSA-2. Forwards were faster than Defensemen in both the tests, however, the difference was significant (p < 0.05) only in RSSA-2. In Forwards and Defensemen, HR_aver_ increased gradually from set 1 through set 6 in RSSA-2 and RSSA-3. In most of the sets, RPE was higher in RSSA-2 than in RSSA-3, and Defensemen perceived higher exertion than Forwards. No difference in [BLa] was noted between Forwards and Defensemen, although players of both positions showed higher [BLa] in RSSA-3 than in RSSA-2. This study concludes that (1) Forwards skate faster than Defensemen, (2) average heart rate and [BLa] do not vary between Forwards and Defensemen, and (3) a higher perceived exertion is observed in Defensemen than Forwards during repeated sprint skating tests

## Introduction

As a team sport, the game of ice hockey is distinctive in many ways. Frequent substitutions of players is one of the characteristic features of ice hockey. Ice hockey players alternate at a regular interval, known as “shifts” to maintain a very fast pace and to regenerate depleted energy stores (Brocherie et al., 2018). Each shift varies in duration from 30 to 80 s, with 2 to 5 min of passive recovery between two consecutive shifts (Brocherie et al., 2018). Duration of the passive recovery or between shifts is determined by the coach and largely based on the match strategy as well as the fitness profile of players. Athletes engaged in team sports sprint repeatedly with brief pauses, consisting of rest and moderate-intensity activity. Although sprints constitute a small part of the game (1-10% of the total distance covered), they play a decisive role in the match result (McGawley and Bishop, 2008; Parolin et al., 1999).

Several repeated sprint ability (RSA) tests have been designed for team sport athletes based on the demand of an intensive period of match play (Bishop et al., 2001; Da Silva et al., 2010; Girard et al., 2011; Hůlka et al., 2014; Rampinini et al., 2009). Repeated sprint ability tests are useful for determining and monitoring anaerobic endurance of athletes, thus delaying early onset of fatigue (Girard et al., 2011). The simplicity and reproducibility of the RSA tests are significant reasons for their popularity. Many researchers have investigated the physiological demands of ice hockey. However, to the best of our knowledge, the effect of recovery duration in ice hockey match play, or an appropriately designed RSA test in ice hockey has not yet been determined. An optimal recovery period between shifts of the game is a challenging issue for coaches and scientists.

Based on the movement patterns of ice hockey players during a competitive match, conditioning coaches and sports scientists have designed repeated sprint skating ability (RSSA) tests (Hůlka et al., 2014; Steeves and Campagna, 2019). In the present study, we compared the effects of two different recovery periods of 2 min and 3 min, between bouts or sets of RSSA, on sprint performance and psychophysiological responses of ice hockey players. The objectives of this study were (a) to determine the effects of two protocols of RSSA tests with two different rest intervals (2 min and 3 min) between the sets on the sprint skating ability of Forwards and Defensemen, (b) to compare the impact of RSSA on the heart rate (HR), blood lactate concentration ([BLa]), and the rate of perceived exertion (RPE) in Forwards and Defensemen.

## Methods

### Participants

Twenty-four professional ice hockey players, 16 Forwards and 8 Defensemen, from a club in Poland participated in this study. Table 1 presents the age, body height, body mass, and body composition of the study participants. Defensemen had 5 to 11 years and Forwards had 6 to 13 years of ice hockey playing experience. The present study was conducted in the preseason of 2020-2021. All players were involved in a periodized strength and conditioning program before the preseason. Participants were acquainted with the experimental method and potential risks involved in this study. Players or their legal guardians, gave written informed consent before participating in the study. The Ethics Committee of the Jerzy Kukuczka Academy of Physical Education in Katowice, Poland, approved the study (No. 8/2018).

**Table 1:** Physical and physiological characteristics of hockey players (n = 24).

Variables	Forwards (n = 16)	Defensemen (n = 8)	Δ (%)	*p*-value	Effect size
Age	23.4 ± 4.76	22.3 ± 5.20	1.2 (5.1%)	0.582	0.24 / Small
Body height (cm)	179.8 ± 5.68	182.0 ± 3.46	-2.2 (-1.2%)	0.331	0.43 / Small
Body mass (kg)	80.5 ± 7.57	87.1 ± 4.81	-6.6 (-8.2%)	0.036	0.97 / Moderate
Body fat%	14.9 ± 4.75	17.3 ± 3.08	-2.4 (-16.1%)	0.208	0.56 / Small
Muscle mass(kg)	39.2 ± 4.02	41.4 ± 2.19	-2.2 (-5.5%)	0.171	0.61 / Moderate
VO_2max_ (ml∙kg^-1^∙min^-1^)	52.3 ± 3.11	50.7 ± 6.24	1.6 (3.1%)	0.398	0.37 / Small
HR_max_ (bpm)	197.8 ± 13.09	195.4 ± 5.78	----	----	----

*Data are presented as mean* ± *SD. Abbreviations: HR_max_ = maximum heart rate; VO_2max_ = maximum oxygen uptake*

### Design and Procedures

The study was completed in four phases. Body height, body mass, and body composition of players were determined in phase 1. In phase 2, the maximum heart rate (HR_max_) was determined and VO_2max_ was estimated by the skating multistage aerobic test (SMAT). In phases 3 and 4, repeated sprint skating ability of players, using two different rest periods (2 and 3 min), was determined in randomized order. Experimental conditions of phases 2, 3, and 4 were similar, and phases were conducted at the same time of the day (10 a.m. to 1 p.m. and 6 p.m. to 9. p.m.). Players were instructed to abstain from moderate to heavy physical activity 24 h before the experiment and maintain a normal diet and fluid intake during the entire study period.

#### Measurement of body composition

Body height was measured using a stadiometer (Seca 213, Seca GmbH & Co, Hamburg, Germany). Body mass, total muscle mass, fat mass, and total body water were evaluated with the bioelectric impedance method (InBody 220, Biospace, Seoul, South Korea).

#### Skating Multistage Aerobic Test (SMAT)

Leone et al. (2007) designed this on-ice test to predict VO_2max_. A 45 m course, defined with markers at both ends of the ice hockey rink, was used to conduct this test. The player skated from one end to the other with a preset speed, which gradually increased until the player failed to maintain the designated speed. Each player held the hockey stick with the preferred hand while skating. The skating speed was dictated by a calibrated audio player that emitted audible signals. The starting speed was set at 3.5 m∙s^-1^, and was increased step-by-step by 0.2 m∙s^-1^. A rest period of 30 s was allowed before beginning the next stage of the test. The following formula was used to predict VO_2max_ of participants (Leone et al., 2007):


$${VO}_{2\max}=18.07\times \left(\right.\text{maximal velocity in}\left.\;m\cdot s^{-1}\right)-\;35.596\;mL\cdot {kg}^{-1}\cdot {min}^{-1}.$$

#### Warm-up before the Repeated Sprint Skating Ability (RSSA) test

A similar warm-up procedure was followed before the RSSA-2 and RSSA-3. The total duration of the warm-up before each RSSA test was 20 min, and it consisted of a 15 min off-ice and a 5 min on-ice warm-up. A twenty-minute rest interval was given between the off-ice and on-ice warm-up to put on hockey gear.

#### Repeated Sprint Skating Ability (RSSA) test

The test was based on the movement pattern and duration of each sprint of players performed during match play and was originally designed by Hůlka et al. (2014). Each player performed six sets of 3 × 80 m sprint trials. Each repetition of 80 m sprint consisted of 18 m of skating forward straight from the goal line, stopping at the blue line, and then skating backward 22 m to the goal line, followed by skating forward 22 m and then turning right, and finally, skating 18 m forward to finish at the goal line. After completing each repetition, the player skated slowly to the starting line. The rest interval between two consecutive repetitions of 80 m sprints was 30 s, and it included slow skating/gliding from the finishing line to the starting line of the next repetition. The detailed description of the test is presented in the paper of Baron et al. (2021). A photocell automatic laser timing system (Microgate, Race time 2, Bolzano, Italy) was used to evaluate each repetition of sprint skating, whereas recovery time was measured using a stopwatch. Based on the duration of the recovery period between two sets of sprint skating, the player performed two types of RSSA tests, i.e., RSSA-2 and RSSA-3, in randomized order. The recovery period between the two sets of RSSA-2 was 2 min, while the recovery period between two consecutive sets of RSSA-3 was 3 min. The RSSA-2 and RSSA-3 tests were separated by five days.

#### Heart rate recording

A heart rate telemeter, Polar Team 2, was used to record the HR continuously during SMAT. During RSSA tests (RSSA-2 and RSSA-3), the HR was registered until the last withdrawal of the blood sample. The recording interval of the HR in all cases was set at 2 s.

#### Blood lactate concentration measurement

Professional phlebotomists collected finger-prick capillary blood for determining [BLa] by an automated lactate analyzer (Biosen C-Line, EKF Diagnostics, UK). This measures lactate concentration by an enzymatic-amperometric method, using chip-sensor technology. Blood was withdrawn about 1 min after the end of the warm-up and 3 min after the completion of the last (6^th^) set of RSSA-2 and RSSA-3 tests. Blood samples were evaluated for lactate concentration within 6 hours after withdrawal.

#### Rate of perceived exertion

Borg’s CR-10 scale was used to determine the RPE (Borg, 1990). The RPE was recorded at rest, after the end of the warm-up, and following the end of every set of both RSSA-2 and RSSA-3.

### Statistical analysis

The values of all the measured variables were presented as the mean and standard deviation (SD). The Shapiro-Wilk test was used to verify the normal Gaussian distribution of the data. Levene’s and Mauchly’s tests verified homoscedasticity and sphericity of data. To determine differences between Forwards and Defensemen, the Student's *t*-test for independent samples was used for normally distributed data and equal variances. The *t*-Student test with Cochran-Cox adjustment was used for normally distributed data yet with unequal variances, and the U-Mann Whitney test for non-normally distributed data. The effect size (ES) was calculated using Cohen’s guidelines. Threshold values for ES were >0.2 (small), >0.6 (moderate), >1.2 (large), and >2.0 (very large) (Hopkins et al., 2009). A two-way analysis of variance with repeated measures and HSD (Honestly Significant Difference) was used. The Tukey post hoc test was used to investigate differences. In relation to the results obtained based on the Borg's CR-10 scale, Friedman's analysis of variance and Dunn's post hoc tests were used. The relationships between HR_aver_ and RPE and between average speed in the last set of RSSA and post-RSSA blood lactate were determined with Pearson’s product-moment correlation analysis. Statistical significance was set at *p* ≤ .05. Statistica 13.3 (TIBCO Software Inc., Palo Alto, CA, USA) was used to conduct all statistical analyses.

## Results

### Physical and physiological characteristics

Table 1 shows the selected physical and physiological characteristics of the study participants. HR_max_ and VO_2max_ were measured by SMAT. Body fat% was marginally higher (ES = small), whereas VO_2max_ was marginally lower (ES=small) in Defensemen compared to their Forward counterparts.

### RSSA test results

**Figure 1:**
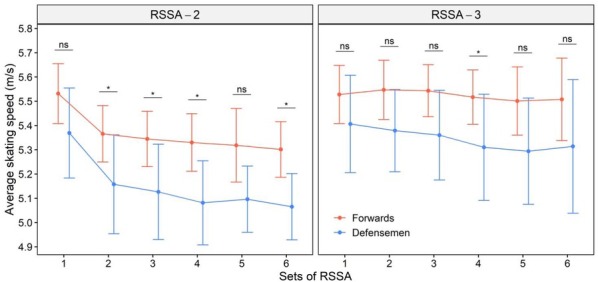
Average skating speed of Forwards and Defensemen in RSSA-2 and RSSA-3 tests (* p < 0.05; ns = non-significant).

Figure 1 shows the average speed of Forwards and Defensemen in RSSA-2 and RSSA-3. Except for Set 1, RSSA-3 was performed significantly faster than the corresponding set of RSSA-2. Forwards completed most of the sets of RSSA-2 (except Sets 1 and 5) significantly (*p* < 0.05) faster than Defensemen. Forwards skated significantly faster than Defensemen in RSSA-2 (except, Sets 1 and 5). A similar trend was noted in RSSA-3, although the difference between Forwards and Defensemen was significant only in Set 4.

### Heart rate response

**Figure 2:**
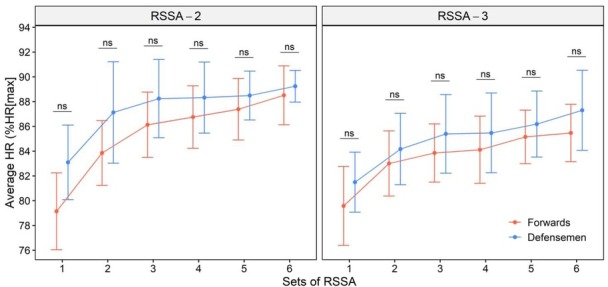
Average heart rate (%HR_max_) of Forwards and Defensemen during RSSA-2 and RSSA-3 tests (ns = non-significant).

The average HR (in terms of %HR_max_) of Forwards and Defensemen in RSSA tests is presented in Figure 2. A slow rise in HR_aver_ from Set 1 was observed in both RSSA-2 and RSSA-3, and the highest HR_aver_ was recorded in the final sets only. There was no significant difference in HR_aver_ between Forwards and Defensemen in RSSA-2 or RSSA-3. RSSA-2 showed a higher heart rate response than in the corresponding set of RSSA-3, in both Forwards and Defensemen, although the difference was not significant in all the sets. However, the HR attained higher values in RSSA-2 than in the corresponding set of RSSA-3, both in Forwards and Defensemen, although the difference was not significant.

### Perceptual response

**Figure 3:**
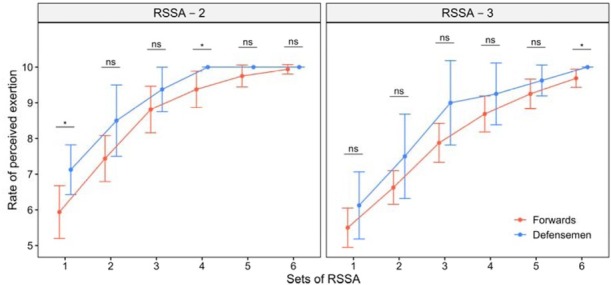
Rate of perceived exertion of Forwards and Defensemen in RSSA-2 and RSSA-3 tests (* p < 0.05; ns = non-significant).

Figure 3 presents the RPE after each set of RSSA-2 and RSSA-3. Although the difference was not always significant between the sets, a gradual increase in the RPE was found in both RSSA-2 and RSSA-3. A steady rise in the RPE with the advancement of sets of RSSA was noted in both RSSA-2 and RSSA-3. The difference was not always significant between RSSA-2 and RSSA-3. However, the RPE was lower in RSSA-3 than RSSA-2 for any given set, but the difference was significant (p < 0.05) in Sets 2, 3, 4, and 5 only. Defensemen perceived higher exertion than Forwards in all the sets, although the difference was significant in one or two sets only.

**Figure 4:**
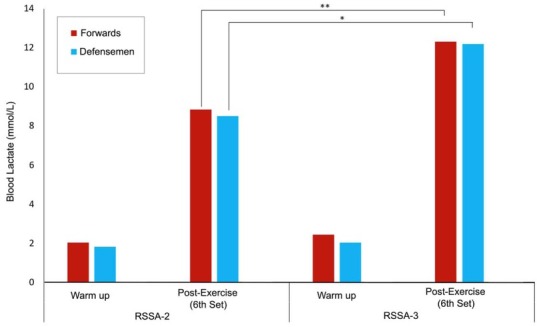
Blood lactate concentrations after the warm-up and following the last set of RSSA-2 and RSSA-3 tests (** p < 0.01; *p < 0.05).

Figure 4 presents the [BLa] after the warm-up and following the end of Set 6 of RSSA tests. Higher [BLa] after RSSA-3 was noticed compared to RSSA-2 in both Forwards and Defensemen. No difference in [BLa] between Forwards and Defensemen was found.

## Discussion

The key findings of this study are that (a) 3 min recovery (i.e., RSSA-3) enhances performance (speed) and causes higher [BLa] in comparison to 2 min recovery (RSSA-2), (b) in both forms of RSSA, Forwards skated faster than Defensemen, (c) HR response was similar in Forwards and Defensemen, and (d) the perceived exertion was higher in Defensemen than in Forwards in most cases.

The game of ice hockey demands a lean body mass to maximize mobility to support sprint, faster change of direction, agility, balance, and frequent high impact body contact (Montgomery, 1988; Twist and Rhodes, 2008). Forwards were reported to have lower body fat (10.8%) than Defensemen (12.1%) and Goalkeepers (13.5%) (Twist and Rhodes, 2008). Participants of this study had higher body fat % than elite hockey players, but the values were similar to top Polish ice hockey players (Roczniok et al., 2016). A higher workload demand of Forwards during match play and training were responsible for the difference in body composition in players of different playing positions (Montgomery, 1988). Superior anaerobic endurance, muscle strength, and power are some of the prerequisites for repeated high-intensity energy output during a game. VO_2max_ of participants in this study were very similar to top Polish ice hockey players (Roczniok et al., 2016), but lower than elite ice hockey players (Burr et al., 2008).

Body fat plays a protective role against injury and protects players from collision during the game. On the contrary, high body fat and weight increase frictional resistance on ice and are likely to reduce skating speed and related performance variables in an ice hockey game (Montgomery, 1988). Slower skating speed in Defensemen, in both RSSA-2 and RSSA-3, can be explained by their higher body fat % and body mass compared to Forwards. Positional differences and differences in training are probably responsible for differences in physical and physiological characteristics of players. Slower movement of Defensemen during real match play is also a common finding by researchers (Green et al., 1976). Despite higher skating speed, HR response was not greater in Forwards, probably due to better cardiovascular conditioning than in Defensemen. Nearly equally high HR_aver_ in Forwards and Defensemen during an ice hockey match was reported by researchers. Ice hockey Forwards and Defensemen were reported to play a match with an HR_aver_ of 161 and 158 b∙min^-1^, respectively, with a peak HR of 195 and 197 b∙min^-1^, respectively (Stanula et al., 2016; Stanula and Roczniok, 2014). The average HR attained in ice hockey players often reaches 85% of their HR_max_, and HR_peak_ often surpasses 90% HR_max_ (Montgomery, 1988; Stanula and Roczniok, 2014). A gradual rise in HR_aver_ and HR_peak_ with the progression of sets of RSSA supports more involvement of the aerobic system at the end stage of RSSA tests. An overall better physiological adaptation and higher training loads may explain the lower RPE (though marginal, in most of the sets of RSSA) in Forwards.

Anaerobic endurance is an important fitness requirement in ice hockey. It allows a player to perform repeated sprints by faster recovery between the bouts of sprints (Girard et al., 2011). Oxidative phosphorylation contributes to under 10% of energy during a short sprint, but with repeated sprint bouts, the contribution of the oxidative system increases gradually. Thus, at the final stage of repeated sprint activity (or RSSA), the aerobic system may contribute up to 40% of the total energy supply (McGawley and Bishop, 2008). The findings of this study clearly indicate that one extra minute of passive recovery significantly improves skating speed and reduces the RPE in both Forwards and Defensemen. The recovery period allows restoration of the anaerobic energy system, yet 2 or 3 min recovery between the sets of RSSA causes only partial restoration of the anaerobic energy substrates. However, it is expected that the resynthesis of energy substrates is significantly higher after 3 min of recovery (i.e., RSSA-3) in comparison to the 2 min recovery period (i.e., RSSA-2). A gradual depletion of anaerobic energy substrates and the increased metabolic acidosis are responsible for the decreased sprinting ability towards the end of the RSSA-2 and RSSA-3 tests. The resynthesis of high energy substrates and acid-base balance are greater after 3 min of recovery.

The game of ice hockey is largely dependent on anaerobic glycolysis as the energy source. This is reflected by the high [BLa] in players at various moments of match play. In competitive ice hockey games, blood lactate contractions vary from 8.2 to 13.7 mmol∙L^-1^ (Noonan, 2010). Movement patterns and recovery periods in this study show some similarities with real match play. Lactate concentrations evaluated after RSSA-2 and RSSA-3 were close to the players’ [BLa] during real match play. Higher [BLa] in RSSA-3 likely results from faster skating by players than in RSSA-2. Despite faster sprints by Forwards, [BLa] was similar to that of Defensemen. It is unknown whether more immediate lactate removal from plasma or reduced lactate production in Forwards are responsible for similar [BLa] as in Defensemen. Repeated sprint performance and its relationship with [BLa] is still unclear (Gaitanos et al., 1993; Girard et al., 2011).

## Conclusions

The results of the present study suggest that (1) both Forwards and Defensemen skate faster when the recovery period is 3 min, instead of 2 min, between sets of repeated sprint skating, (2) no difference in cardiac workload and glycolytic response exists between Forwards and Defensemen during maximum sprint skating, and (3) Defensemen perceive higher exertion than Forwards during sets of repeated sprints that mimic basic movement patterns of an ice hockey game. This study may have practical implications for ice hockey coaches in the effective use of Forwards and Defensemen by selecting suitable bench time for improved speed performance and prevention of early fatigue.
